# Intraoperative Dynamics of Lactate, pH, and Bicarbonate During Cardiac Surgery: A Prospective Study Exploring a Composite Metabolic Marker of Reperfusion Stress

**DOI:** 10.3390/diagnostics16162597

**Published:** 2026-08-16

**Authors:** Andrei Raicea, Liviu Moraru, Victor Raicea

**Affiliations:** 1 Doctoral School of Medicine, George Emil Palade University of Medicine, Pharmacy, Science, and Technology of Târgu Mureș, 540142 Târgu Mureș, Romania; raicea_andrei@yahoo.com; 2 Department of Cardiovascular Surgery, Emergency Institute for Cardiovascular Diseases and Transplantation, 540136 Târgu Mureș, Romania; 3 Department of Anatomy, University of Medicine, Pharmacy, Science and Technology “George Emil Palade” of Târgu-Mureș, 540142 Târgu-Mureș, Romania; 4 Clinic of Cardio-Vascular Surgery, Emergency Institute for Cardiovascular Diseases and Transplantation, 540136 Târgu Mureș, Romania; 5 Department of Cardiovascular Surgery, University of Medicine and Pharmacy Craiova, 200349 Craiova, Romania; victor.raicea@umfcv.ro

**Keywords:** cardiac surgery, mortality, myocardial perfusion index

## Abstract

**Background:** Myocardial ischemia–reperfusion during cardiac surgery is associated with complex metabolic alterations that reflect both myocardial injury and recovery. This prospective study aimed to characterize intraoperative lactate, pH, and bicarbonate dynamics in paired coronary sinus (CS) and peripheral blood samples and to explore whether these responses could be integrated into a composite marker of reperfusion-related metabolic burden (RMB). **Methods:** We prospectively studied 101 patients undergoing cardiac surgery with cardiopulmonary bypass. Simultaneous blood samples from the CS and peripheral circulation were obtained before aortic cross-clamping (T0), immediately after declamping (T1), and 10 min after reperfusion (T2). Temporal changes and transmyocardial gradients of lactate, pH, and bicarbonate were analyzed. RMB was calculated from standardized changes in these variables. As an exploratory analysis, associations with in-hospital mortality were evaluated using receiver operating characteristic (ROC) analysis. **Results:** Significant temporal variations in metabolic parameters were observed, with the most pronounced transmyocardial disturbances occurring at aortic declamping. Lactate demonstrated the largest gradient during early reperfusion, accompanied by transient acidosis and bicarbonate consumption. The RMB framework integrated these coordinated metabolic responses into a single measure of reperfusion stress. In exploratory outcome analyses, higher RMB values were observed among non-survivors, with the largest observed AUC for peripheral RMB measured 10 min after reperfusion (AUC 0.87). However, these estimates were based on only seven deaths and should be considered hypothesis-generating. **Conclusions:** Paired CS and peripheral sampling revealed dynamic metabolic adaptations during myocardial ischemia–reperfusion. The exploratory RMB framework integrates coordinated metabolic changes into a single descriptive measure of reperfusion-related stress. Its observed associations with in-hospital mortality remain preliminary and require confirmation through external validation and evaluation in larger independent prospective cohorts.

## 1. Introduction

Despite substantial advances in surgical techniques and myocardial protection strategies, ischemia–reperfusion injury remains a major contributor to postoperative morbidity and mortality in cardiac surgery [1,2,3]. During cardiopulmonary bypass (CPB) and aortic cross-clamping, the myocardium undergoes controlled ischemia followed by abrupt reperfusion, which induces significant metabolic and biochemical changes. The severity of these changes reflects both the degree of ischemic and reperfusion stress and the efficacy of myocardial protection strategies. Beyond ischemic duration, the metabolic response to reperfusion is influenced by cardioplegia and reperfusion strategies, oxygen delivery during cardiopulmonary bypass, and the capacity of the myocardium to restore oxidative metabolism [4,5,6,7,8,9]. Early identification of patients experiencing severe myocardial metabolic injury during reperfusion remains clinically important, as it may allow improved risk stratification and prompt optimization of perioperative management.

Current predictors of adverse outcomes in cardiac surgery include operative variables such as aortic cross-clamp and cardiopulmonary bypass duration, as well as biochemical markers including cardiac troponin and systemic lactate concentrations [10,11,12,13,14,15,16]. However, these variables provide only indirect information regarding myocardial metabolic injury. Operative duration reflects procedural complexity and cumulative exposure to ischemic and systemic stress, whereas systemic lactate concentrations are influenced by oxygen delivery–consumption imbalance, hemodilution, cardiopulmonary bypass duration, perfusion pressure, microcirculatory dysfunction, impaired mitochondrial respiration, and extracardiac lactate production [13,14,17,18,19,20,21,22,23,24]. Consequently, these variables may fail to capture the dynamic metabolic processes occurring within the myocardium during early reperfusion.

Therefore, direct evaluation of myocardial metabolic activity could provide a more physiologic understanding of ischemia–reperfusion injury. Blood collected from the coronary sinus (CS) represents the venous return from the myocardium, allowing evaluation of transmyocardial metabolic exchanges during cardiac surgery. Previous studies have shown that CS lactate release reflects myocardial anaerobic metabolism during ischemia. Previous clinical studies using coronary sinus or paired coronary venous–arterial sampling have demonstrated early myocardial lactate release, changes in coronary venous acid–base status, delayed recovery of lactate extraction, and concomitant release of myocardial injury biomarkers during reperfusion [7,8,25,26,27]. However, isolated metabolic markers provide only a partial representation of the complex metabolic disturbances that accompany reperfusion after ischemia [28,29].

Although lactate, pH, and bicarbonate each provide information regarding myocardial metabolic status, they reflect different and complementary aspects of ischemia–reperfusion physiology, including anaerobic metabolism, acid–base disturbance, and buffering capacity. Simultaneous assessment of these variables from paired CS and peripheral samples may therefore provide a more comprehensive characterization of myocardial metabolic adaptation during reperfusion than any individual parameter alone.

Despite extensive investigation of myocardial protection strategies, detailed characterization of transmyocardial metabolic changes during the earliest phases of reperfusion remains limited, particularly using paired coronary sinus and peripheral sampling. Accordingly, we conducted a prospective study to characterize intraoperative lactate, pH, and bicarbonate dynamics during cardiac surgery with cardiopulmonary bypass. As a secondary exploratory objective, we evaluated whether standardized temporal changes in these metabolic variables could be integrated into a composite marker of reperfusion-related metabolic burden (RMB) and explored its association with in-hospital mortality.

## 2. Objectives

The primary objective of this study was to characterize transmyocardial and systemic changes in lactate, pH, and bicarbonate during myocardial ischemia and early reperfusion in patients undergoing cardiac surgery with cardiopulmonary bypass. A secondary exploratory objective was to investigate whether these metabolic responses could be integrated into a composite marker of reperfusion-related metabolic burden and to examine its association with in-hospital mortality.

## 3. Material and Methods

### 3.1. Population

This prospective, single-center observational study included 101 consecutive adult patients who underwent cardiac surgery with cardiopulmonary bypass and aortic cross-clamping between 2021 and 2024, including four emergency procedures. All operations were performed by the same surgical team. The inclusion criteria were age > 18 years; absence of significant coronary artery disease, confirmed by coronary angiography performed within 3 months before surgery; availability of a complete metabolic sampling protocol; a cardiac procedure requiring bicaval venous cannulation, total cardiopulmonary bypass, and right atriotomy to permit direct coronary sinus sampling; and absence of pre-existing severe metabolic acidosis.

The study protocol was approved by the Ethics Committee of the University of Medicine and Pharmacy of Craiova (Approval No. 74, 7 September 2020). The study was conducted in accordance with the Declaration of Helsinki, and written informed consent was obtained from all participants before enrollment.

### 3.2. Intraoperative Management

All procedures were performed under standardized general anesthesia for cardiac surgery. Total CPB was established using non-pulsatile flow. To collect blood from the coronary sinus, patients required bicaval venous cannulation, snaring of the venae cavae, and a right atriotomy for direct sampling. Patients underwent moderate hypothermia (28 degrees C). Myocardial protection was achieved using antegrade standardized cardioplegia protocols. Cardioplegic solution with potassium-enriched blood from the pump was administered every 20–30 min during cardiac ischemia in all patients (Calafiore-type cardioplegia).

Ischemic time (IT) and total bypass time (TBP) were recorded for each patient.

### 3.3. Metabolic Sampling Protocol

Simultaneous blood samples were obtained from the peripheral cardioplegic line (P) and from the CS. The samples were obtained during the following time points:

• Preclamp (T0): Pre-clamping of the aorta (baseline metabolic state);
• Declamp (T1): Immediately after declamping in the first 30 s (onset of reperfusion);
• 10declamp (T2): 10 min after declamping (early reperfusion phase).

The following parameters were measured at each time point:

• Lactate (mmol/L);
• pH;
• Standard bicarbonate (HCO_3_^−^, mmol/L).

All analyses were performed using the same blood gas analyzer to minimize inter-assay variability. Two distinct types of metabolic differences were analyzed in this study: transmyocardial gradients and temporal changes. Ischemic time (IT) and total bypass time (TBP) were recorded for each patient.

Transmyocardial metabolic gradients were calculated as the difference between CS and peripheral cardioplegic line measurements for all parameters at each time point. These gradients quantify myocardial-specific metabolic activity by isolating the heart’s contribution from the systemic circulation.

Temporal changes (Δ) were defined as differences between consecutive time points and were used for the calculation of the Reperfusion Metabolic Burden (RMB) index.

For each metabolic parameter X, temporal changes were calculated between consecutive predefined time points:

$$\Delta X_{T1}=X_{T1}-X_{T0}$$

$$\Delta X_{T2}=X_{T2}-X_{T1}$$

where T0 represents the preclamp value, T1 the value immediately after aortic declamping, and T2 the value 10 min after declamping.

### 3.4. *Standardization of Variables*

Z-scores were calculated for each variable based on the change (Δ) from baseline, not the absolute values, to standardize inter-individual variability and enable integration into a composite index:

• Z(ΔXT) = (ΔXT −μΔXT)/σΔXT

where:

• ΔX = individual change in the variable;
• μ = mean change of the cohort;
• σ = standard deviation of the change.

The Reperfusion Metabolic Burden (RMB) index was defined as:

RMBT = Z(ΔLactateT) − Z(ΔpHT) − Z(ΔHCO3 − T)

where T is either T1 or T2.

RMB was calculated at two predefined time points at T1 and T2. By construction, the subtraction terms ensure that all components contribute in the same direction toward metabolic stress. A positive RMB reflects greater lactate accumulation, a larger decrease in pH, and increased bicarbonate consumption, consistent with a higher metabolic burden during reperfusion. An RMB value close to zero indicates a metabolic response comparable to the cohort mean, whereas higher positive values reflect a more pronounced reperfusion-related metabolic disturbance.

The three standardized components were assigned equal weights in this initial exploratory formulation of RMB. This choice was based on the complementary physiological domains represented by lactate, pH, and bicarbonate: lactate reflects anaerobic metabolism and metabolite washout, pH reflects the overall acid–base disturbance, and bicarbonate reflects buffering consumption and recovery. Z-score standardization placed the variables on a common scale, allowing each component to contribute comparably despite their different units and numerical ranges. Equal weighting was selected to preserve interpretability and to avoid deriving outcome-dependent weights from the same relatively small cohort in which the index was evaluated, which could have increased the risk of overfitting. The weighting strategy was therefore heuristic and physiologically informed rather than statistically optimized.

### 3.5. Statistical Analysis

Continuous variables were assessed for normality using the Shapiro–Wilk test and are presented as mean ± standard deviation (SD) or median (interquartile range [IQR]), as appropriate. Normally distributed variables were compared using the independent-samples Student’s t-test, whereas non-normally distributed variables were compared using the Mann–Whitney U test. Categorical variables are presented as counts and percentages and were compared using the χ^2^ or Fisher’s exact test, as appropriate. Pearson correlation was used for normally distributed variables and Spearman rank correlation for non-normally distributed variables.

No formal a priori sample size or power calculation was performed. The study included all consecutive eligible patients with complete paired metabolic measurements during the predefined study period. Consequently, analyses involving in-hospital mortality were considered exploratory and were not powered to establish definitive prognostic performance.

Associations between RMB and clinical outcomes were assessed using correlation analysis. Discriminative performance was evaluated using receiver operating characteristic (ROC) analysis and the area under the curve (AUC), with the optimal cut-off determined by the Youden index. Because this was an exploratory study evaluating multiple metabolic variables, sampling compartments, time points, and outcomes, no adjustment for multiple comparisons was applied. Therefore, all reported *p*-values are nominal and should be interpreted as descriptive and hypothesis-generating rather than confirmatory. Given the limited number of deaths, no multivariable prognostic model was developed, and all ROC analyses were interpreted descriptively.

To assess the internal stability of the exploratory ROC analyses, stratified nonparametric bootstrap resampling (2000 iterations) was performed for the two RMB measurements with the highest apparent discrimination. Resampling was conducted separately within the survivor and non-survivor groups to preserve the observed outcome distribution. Within each bootstrap sample, the z-score standardization parameters were recalculated, the RMB index was reconstructed, and the AUC was estimated. Bootstrap-derived 95% confidence intervals and optimism-corrected AUCs were calculated.

Formal subgroup analyses according to surgical procedure were not performed because the procedural categories were unbalanced and several subgroups contained few patients. In addition, the small number of in-hospital deaths precluded reliable outcome analyses within individual procedural subgroups.

Serial metabolic measurements were analyzed using linear mixed-effects models, with time entered as a repeated within-subject factor and patient as a random effect to account for within-subject correlation. Survival status and the time × survival interaction were included as fixed effects to evaluate differences in temporal trajectories between survivors and non-survivors. This approach accommodates unbalanced repeated measurements and allows inclusion of all available observations without requiring complete data at every time point.

### 3.6. Use of Generative Artificial Intelligence

OpenAI ChatGPT (GPT-5.6 Thinking; OpenAI, San Francisco, CA, USA; accessed July 2026) was used during manuscript revision to assist with English-language editing, structural refinement, and the drafting and rephrasing of selected passages and responses to peer-review comments. The tool was not used to generate or modify study data or to perform the statistical analyses. All AI-assisted content was critically reviewed, verified, and edited by the authors, who take full responsibility for the final content of the manuscript.

## 4. Results

Unless otherwise specified, the *p*-values reported in this section are nominal and were not adjusted for global multiple testing.

### 4.1. Population and Surgery Characteristics

The study group included 101 adult patients undergoing cardiac surgery with extracorporeal circulation. The mean age was 59.5 ± 10.8 years, and 63 patients (62.4%) were male. The overall mortality was 6.9% (7/101). All deaths occurred during hospital admission (Table 1).

**Table 1:** Population characteristics.

**Population Characteristics**	**N** **= 101**
Age (years)	59.5 ± 10.8
Age median	61 [54–67.5]
Gender (males)	63/101 (62.4%)
Deaths	7/101 (6.9%)
**Surgical characteristics**	
Type of surgery	
Isolated AVR	21 (21%)
Isolated MVR	41 (41%)
AVR + MVR	29 (29%)
MVR + TV plasty	7(6.9%)
Other	3(3%)
Bypass time (min)	97.2 ± 64.5 (interval 27–410)
Ischemic time (min)	61.5 ± 39.0 (interval 15–189)

AVR, aortic valve replacement, MVR, mitral valve replacement, TV, tricuspid valve.

### 4.2. Metabolic Characteristics

CS lactate levels were significantly higher than peripheral levels at all sampling time points (all *p* < 0.001) (Table 2).

**Table 2:** Average lactate values at different time points.

Lactate (mmol/L)	Peripheral	Coronary Sinus	*p* Value
T0	1.21 ± 0.62	1.57 ± 0.64	*p* < 0.0001
T1	2.44 ± 0.96	4.66 ± 2.15	*p* < 0.0001
T2	1.82 ± 0.68	2.33 ± 0.8	*p* < 0.0001

Coronary sinus pH was significantly lower at T1 (*p* < 0.001) and T2 (*p* < 0.001) compared to peripheral sampling, whereas no difference was observed at baseline (*p* = 0.16).

Average pH and bicarbonate values at different time points may be observed in Table 3 and Table 4.

**Table 3:** Average pH values at different time points.

pH	Peripheral	Coronary Sinus	*p* Value
T0	7.44 ± 0.06	7.43 ± 0.04	*p* = 0.16
T1	7.42 ± 0.07	7.32 ± 0.09	*p* < 0.0001
T2	7.44 ± 0.06	7.41 ± 0.05	*p* < 0.001

Standard bicarbonate levels differed significantly between compartments at T1 (*p* < 0.001), whereas no significant differences were observed at T0 or T2.

**Table 4:** Average bicarbonate values at different time points.

Bicarbonate (HCO_3_^−^)	Peripheral	Coronary Sinus	*p* Value
T0	26.3 ± 3.2	27.3 ± 3.5	*p* = 0.37
T1	20.4 ± 3.4	21.2 ± 2.8	*p* < 0.001
T2	19.4 ± 3.9	22.4 ± 2.5	*p* = 0.10

### 4.3. Transmyocardial Metabolic Gradients

Lactate gradients were significantly higher at T1 compared with both T0 and T2 (both *p* < 0.001), while no difference was observed between T0 and T2 (*p* = 0.09). Similar patterns were observed for pH gradients. In contrast, bicarbonate gradients increased later during reperfusion.

The lactate gradient differed markedly across time points (F = 64.6, *p* < 0.001), as did the pH gradient (F = 34.8, *p* < 0.001). The bicarbonate gradient also varied significantly over time, although with a smaller effect size (F = 7.66, *p* = 0.0006) (Table 5).

**Table 5:** Metabolic gradients.

Transmyocardial Gradient (CS–P) (Mean ± SD)	T0	T1	T2
Lactate (mmol/L)	0.36 ± 0.52	2.22 ± 1.89 *β	0.51 ± 0.64
pH	−0.01 ± 0.03	−0.10 ± 0.07 *β	−0.03 ± 0.04
HCO_3_^−^ (mmol/L)	1.03 ± 2.91	0.79 ± 2.46 β	3.08 ± 2.74 *

* *p* < 0.001 compared to preclamp; β *p* < 0.001 compared to 10 post-declamp.

### 4.4. RMB Index

Table 6 shows the average RMB values calculated from the CS and P. As expected from z-score normalization, mean RMB values were centered around zero. However, substantial inter-individual variability was observed, with standard deviations ranging from 1.9 to 2.3, reflecting heterogeneity in metabolic response to reperfusion.

**CARDIOPULMONARY BYPASS TIME AND TOTAL ISCHEMIC TIME**

The CPB time was 97.2 ± 64.5 min while the ischemic time was 61.5 ± 39.0 min. CPB duration was longer in patients who died compared to survivors (177.4 ± 128.5 min vs. 101.2 ± 62 min, *p* = 0.04).

RMB from the CS at T1 showed weak-to-moderate correlations with the ischemic time (ρ = 0.28, *p* = 0.007) and CPB time (ρ = 0.25, *p* = 0.015). Peripheral RMB at T1 correlated slightly more strongly with both ischemic time (ρ = 0.33, *p* = 0.001) and CPB time (ρ = 0.32, *p* = 0.002), whereas RMB from the CS at T2 was not associated with either operative duration.

**EXPLORATORY ASSOCIATIONS WITH IN-HOSPITAL MORTALITY**

### 4.5. Total Ischemic Time and CPB Time

CPB duration showed a moderate association with mortality (Spearman ρ = 0.23, *p* = 0.021) and demonstrated modest discriminative performance (AUC = 0.72, 95% CI 0.59–0.92, sensitivity 100%, specificity 49%). Ischemic time showed a weaker association (ρ = 0.17, *p* = 0.083; AUC = 0.70, 95% CI 0.54–0.87, sensitivity 100%, specificity 50%).

### 4.6. Metabolic Gradients

Exploratory AUC estimates were low for the metabolic gradients at T0 and at T2 (AUC between 0.35–0.42). At T1, the lactate gradient yielded the largest observed AUC (AUC ≈ 0.78, 95% CI 0.63–0.91).

### 4.7. RMB Index

RMB values in the coronary sinus at T1 were significantly higher in non-survivors compared with survivors (*p* = 0.021). Also, peripheral RMB values at T2 showed the strongest association with mortality (*p* = 0.006). Other RMB measurements were not significantly associated with the outcome (Table 7).

**Figure 1:**
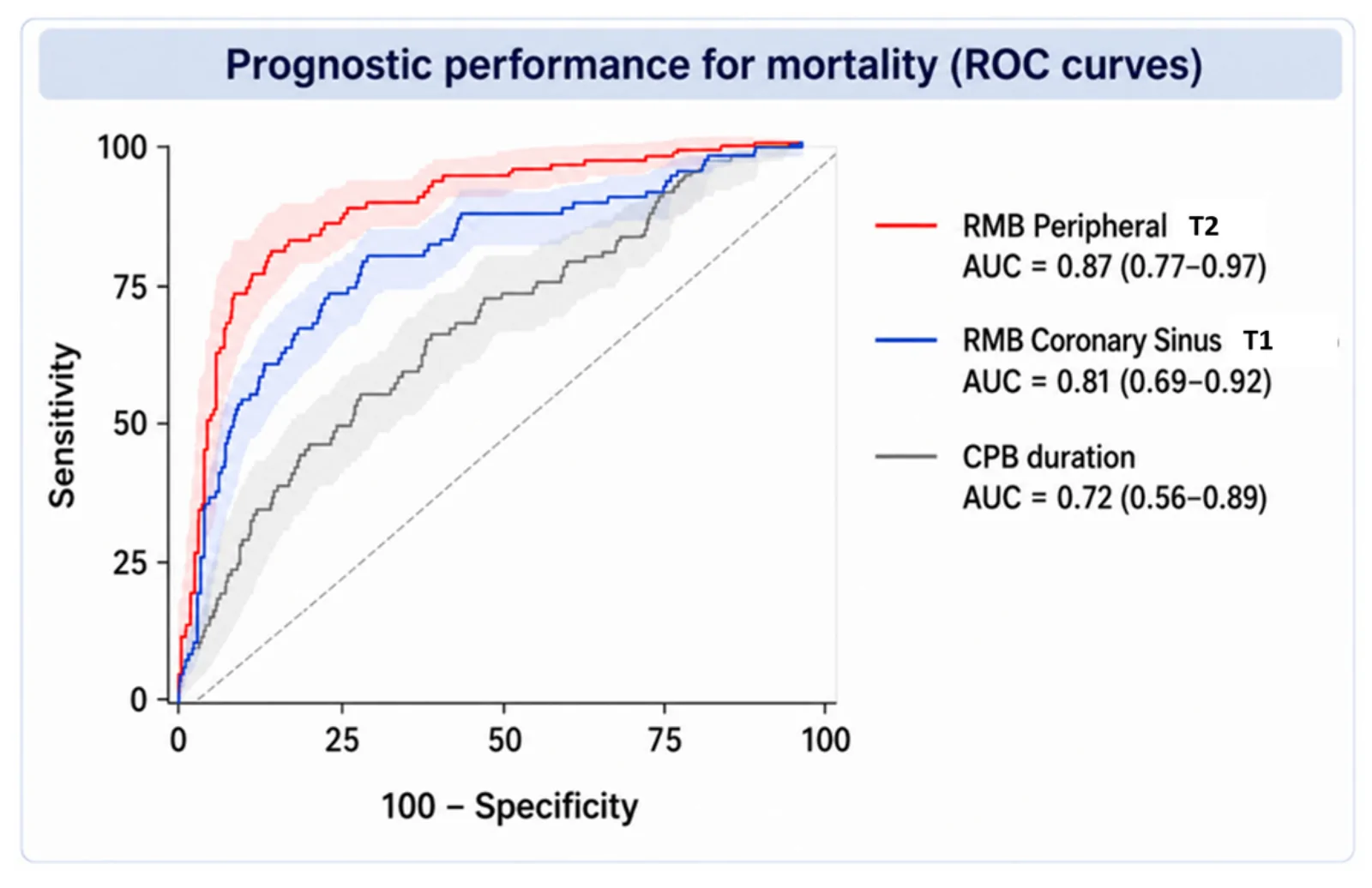
Exploratory receiver operating characteristic (ROC) curves for RMB indices derived from coronary sinus and peripheral samples. Coronary sinus RMB at T1 and peripheral RMB at T2 yielded the largest observed AUC values in this cohort. Because only seven in-hospital deaths occurred, these findings should be interpreted as descriptive and hypothesis-generating rather than as evidence of validated prognostic performance.

In exploratory ROC analysis, CS RMB at T1 and peripheral RMB at T2 yielded AUC values of 0.81 (95% CI 0.69–0.92) and 0.87 (95% CI 0.77–0.97), respectively (Figure 1). These estimates were based on only seven mortality events and are therefore reported descriptively.

### 4.8. Internal Bootstrap Validation

Stratified bootstrap validation yielded an apparent AUC of 0.820 for CS RMB at T1 (bootstrap 95% CI 0.688–0.932), with an estimated optimism of 0.001 and an optimism-corrected AUC of 0.819. For peripheral RMB at T2, the apparent AUC was 0.866 (bootstrap 95% CI 0.775–0.945), with an estimated optimism of <0.001 and an optimism-corrected AUC of 0.866. Despite the minimal estimated optimism, the limited number of mortality events resulted in considerable uncertainty, and these findings remain exploratory.

## 5. Discussion

### 5.1. Main Findings

The present study provides a detailed characterization of metabolic changes occurring during myocardial ischemia and early reperfusion in cardiac surgery using paired coronary sinus and peripheral blood sampling. Three principal observations emerged. First, aortic declamping was associated with marked transmyocardial metabolic disturbances characterized by lactate release, acidosis, and alterations in bicarbonate handling. Second, these responses evolved rapidly during the first minutes of reperfusion, suggesting dynamic metabolic recovery and buffering. Third, integration of these metabolic responses into the exploratory RMB framework provided a composite description of reperfusion-related metabolic stress and showed preliminary associations with in-hospital mortality. Given the limited number of outcome events, these associations should be regarded as hypothesis-generating.

### 5.2. Pathophysiologic Concept

Effective myocardial protection during cardiopulmonary bypass is essential to preserve metabolic stability and limit ischemia–reperfusion injury. During aortic cross-clamping, interruption of coronary perfusion forces cardiomyocytes to shift from oxidative phosphorylation to anaerobic glycolysis, leading to lactate and hydrogen ion accumulation, intracellular acidosis, and impaired energy-dependent ion homeostasis.

Upon aortic declamping, restoration of coronary blood flow triggers rapid washout of these metabolites and re-exposure to oxygen, initiating the biochemical cascade of reperfusion injury. This early reperfusion phase represents a critical metabolic transition, during which the myocardium must re-establish aerobic metabolism and intracellular homeostasis. The magnitude of metabolic disturbance during this period reflects the balance between ischemic injury and the effectiveness of myocardial protection. Human studies have shown that metabolic abnormalities detected in coronary venous blood are most pronounced immediately after restoration of coronary flow and may persist during the initial reperfusion period, reflecting delayed recovery of oxidative substrate utilization [7,8,25,26,27].

In this context, integrating coronary sinus and peripheral metabolic measurements allows a direct assessment of myocardial-specific metabolic activity and its systemic consequences during reperfusion, providing a physiologically relevant signal of early metabolic stress [29].

### 5.3. Role of Oxygenation Strategy and Systemic Determinants of Metabolic Stress

Although reperfusion is essential to restore myocardial viability after aortic cross-clamping, the conditions under which reperfusion is initiated may substantially influence the magnitude of ischemia–reperfusion injury. Experimental and clinical evidence suggests that hyperoxic reperfusion may exacerbate oxidative stress through excessive reactive oxygen species generation, contributing to mitochondrial dysfunction, endothelial injury, calcium overload, and impaired recovery of myocardial contractile function. Conversely, controlled normoxic reperfusion has been proposed as a more physiological strategy capable of attenuating oxidative injury while preserving adequate oxygen delivery. Recent clinical data by El Dsouki et al. [30] demonstrated that patients undergoing coronary artery bypass grafting managed with normoxic reperfusion exhibited lower oxidative stress, reduced perioperative myocardial injury, improved postoperative ventricular function, and shorter intensive care and hospital stay compared with conventional hyperoxic reperfusion. These findings support the concept that oxygenation strategy itself may influence the metabolic response observed during the earliest phase of reperfusion.

At the same time, the present findings should not be interpreted as indicating that lactate release exclusively reflects myocardial reperfusion injury. Hyperlactatemia during cardiopulmonary bypass represents the integrated consequence of multiple physiological processes, including systemic oxygen delivery and consumption, cumulative oxygen debt, hemodilution, cardiopulmonary bypass duration, perfusion pressure, vasoactive support, inflammatory activation, extracardiac lactate production, microcirculatory dysfunction, and impaired mitochondrial respiration [4,5,6,13,14,17,18,19,20,21,22,23,24,28,31]. Goal-directed perfusion studies have shown that maintaining adequate indexed oxygen delivery during cardiopulmonary bypass can reduce postoperative organ injury, supporting the physiological relevance of oxygen delivery–consumption balance when interpreting intraoperative lactate kinetics [4,5,6,17]. Recent studies have also associated postoperative lactate accumulation and lactic acidosis with impaired microvascular perfusion, reduced mitochondrial respiration, acute organ injury, and multiple organ dysfunction [14,19,20,21,22,23]. Consequently, the metabolic alterations observed in the coronary sinus should be interpreted within the broader context of the systemic metabolic response to cardiopulmonary bypass rather than as isolated markers of myocardial injury alone.

This concept is consistent with the recently proposed Lactate Risk Score (LRS) by Condello et al. [31], an evidence-based framework designed to anticipate the development of hyperlactatemia during cardiopulmonary bypass by integrating preoperative characteristics, perfusion adequacy, cumulative oxygen debt, cardiopulmonary bypass duration, hemodynamic stability, vasoactive support, and metabolic variables. However, the LRS requires prospective validation before clinical adoption. Unlike the LRS, which estimates the likelihood of developing systemic hyperlactatemia before it occurs, the exploratory RMB framework was conceived to characterize the immediate metabolic consequences of reperfusion by integrating simultaneous temporal changes in lactate, pH, and bicarbonate obtained from paired coronary sinus and peripheral blood sampling. Therefore, the two approaches should be regarded as complementary rather than competing: the LRS focuses on identifying patients at increased metabolic risk before hyperlactatemia develops, whereas RMB aims to describe the magnitude of the metabolic response once reperfusion has been established.

### 5.4. Metabolic Characteristics and Metabolic Gradients

In our study, lactate concentrations were consistently higher, and pH was consistently lower in the CS than in the peripheral circulation at all sampled time points. This metabolic profile is likely the result of the interruption of oxidative metabolism during aortic cross-clamping, which enables anaerobic glycolysis in cardiomyocytes, leading to the accumulation of lactate and hydrogen ions. With the restoration of coronary blood flow, these metabolites are washed out into the coronary sinus, leading to higher lactate concentrations and lower pH in coronary venous blood compared with the peripheral circulation. This interpretation is consistent with previous clinical studies showing immediate coronary sinus lactate washout, transient coronary venous acidosis, delayed recovery of lactate extraction, and myocardial biomarker release after restoration of coronary flow [7,8,25,26,27].

Lactate exhibited the most pronounced and persistent transmyocardial gradient, with a marked increase in coronary sinus concentrations at the time of declamping, reflecting the rapid washout of metabolites accumulated during ischemia. Although partial normalization occurred within the first 10 min of reperfusion, lactate levels remained elevated, indicating incomplete recovery of oxidative metabolism. In parallel, pH demonstrated a transient but significant myocardial acidosis at the onset of reperfusion, followed by rapid partial normalization, suggesting effective hydrogen ion buffering. In contrast, bicarbonate displayed a distinct temporal pattern: an initial decrease at declamping, reflecting acute buffer consumption, followed by divergence between compartments during early reperfusion, with partial recovery in the coronary sinus and continued decline in the peripheral circulation. Nevertheless, peripheral lactate kinetics also reflect perfusion pressure, global oxygen delivery, microcirculatory flow, mitochondrial function, and extracardiac lactate production, which limits attribution of systemic metabolic changes exclusively to the myocardium [4,5,6,13,14,17,18,19,20,21,22,23,24].

### 5.5. Exploratory Associations with In-Hospital Mortality

The in-hospital mortality rate in the present cohort was 6.9%. Direct comparison with previously reported cardiac surgery populations is difficult because definitions of operative mortality, procedural case mix, urgency, baseline risk, and institutional characteristics vary across studies [32]. Given the limited number of events, no reliable inference can be made regarding the contribution of any individual surgical procedure to the observed mortality rate.

### 5.6. Cardiopulmonary Bypass Time and Total Ischemic Time

In this cohort, longer CPB and ischemic times showed exploratory univariable associations with in-hospital mortality. However, because only seven deaths occurred, the magnitude and stability of these associations cannot be estimated reliably. Operative duration remains clinically relevant as an indicator of procedural complexity and systemic exposure, but it does not directly quantify the myocardial biological response to ischemia–reperfusion or interindividual differences in metabolic recovery. Contemporary studies likewise indicate that longer ischemic and cardiopulmonary bypass exposure and greater procedural complexity may be associated with increased postoperative myocardial biomarker release, while definitions and clinically meaningful thresholds for perioperative myocardial injury remain heterogeneous [15,16].

### 5.7. Influence of Procedural Heterogeneity

The cohort included patients undergoing isolated aortic valve replacement, isolated mitral valve replacement, combined aortic and mitral valve replacement, mitral valve replacement with tricuspid valve repair, and other procedures. Although anesthesia, cardiopulmonary bypass, temperature management, and antegrade blood cardioplegia protocols were standardized, the type of surgical procedure may still influence perioperative metabolic responses. Potential contributing factors include differences in the underlying cardiac pathology, preoperative ventricular remodeling and loading conditions, ischemic and cardiopulmonary bypass duration, the extent of intracardiac manipulation, and perioperative hemodynamic requirements. Procedure-specific metabolic responses may also be modified by the cardioplegia and reperfusion strategy, as suggested by clinical studies evaluating blood versus crystalloid cardioplegia, warm cardioplegia, antioxidant reperfusion strategies, and ischemic postconditioning [7,8,9,22].

The inclusion of several procedural categories broadens the physiological scope of the study but may also obscure procedure-specific metabolic patterns. Subgroup analyses were considered; however, the procedural groups were unbalanced, and some categories included very few patients. In addition, only seven in-hospital deaths occurred in the entire cohort. Inferential analyses within procedural subgroups would therefore have been severely underpowered and vulnerable to unstable or spurious findings. Procedure-specific metabolic trajectories should be examined in larger cohorts designed and adequately powered for such comparisons.

### 5.8. Metabolic Characteristics and Metabolic Gradients

Exploratory ROC estimates for metabolic gradients measured before aortic cross-clamping and 10 min after reperfusion were low, with AUC values ranging from 0.35 to 0.42. Preclamping measurements primarily represent baseline metabolic conditions, whereas measurements obtained at T2 may reflect partial metabolic normalization after restoration of coronary perfusion and activation of systemic buffering mechanisms. These time points may therefore be less sensitive to the immediate metabolic disturbance associated with the onset of reperfusion.

Among the individual metabolic gradients, the lactate gradient at T1 yielded the largest observed AUC of approximately 0.78. Given the limited number of mortality events, this result should be interpreted as a preliminary signal rather than as evidence of predictive validity. Nevertheless, the finding is physiologically consistent with the rapid washout of metabolites accumulated during ischemia. The variable performance of isolated metabolic parameters further illustrates the complex interaction among substrate release, acid–base imbalance, systemic perfusion, and buffering dynamics during early reperfusion.

### 5.9. RMB Index

RMB measured in the coronary sinus at T1 and in peripheral blood at T2 was higher among non-survivors than among survivors. These findings suggest that a more pronounced metabolic disturbance during immediate myocardial reperfusion and persistent systemic metabolic stress during early reperfusion may be associated with an adverse postoperative course. However, the observed differences were based on only seven deaths and should be interpreted cautiously.

In exploratory ROC analyses, CS RMB at T1 and peripheral RMB at T2 yielded AUC values of 0.81 and 0.87, respectively. These values indicate a potential signal within the present cohort but do not establish validated prognostic discrimination. The estimates are likely to be imprecise and potentially optimistic because the index was developed and evaluated in the same cohort and because of the small number of outcome events.

The comparison between RMB and conventional operative variables should therefore not be interpreted as demonstrating superiority of RMB over ischemic time or CPB duration. Rather, the weak correlations between RMB and operative duration suggest that the composite index may capture metabolic information that is not entirely represented by procedural time alone. Confirmation through external validation and subsequent evaluation in independent cohorts is required before any prognostic or clinical interpretation can be made.

Equal weighting was intentionally used for the initial development of RMB to maintain a transparent and readily interpretable composite measure. However, this approach should not be considered the only or necessarily the optimal method of index construction. Alternative strategies could include principal component or factor analysis, regression-derived weighting, penalized models, or weights established through external physiological and clinical evidence. Such methods may better characterize the relative contribution of each component but require larger datasets and independent validation to limit overfitting. Future studies should also examine the degree of shared information between pH and bicarbonate and determine whether all three components provide independent incremental value.

### 5.10. Study Implications and Novelty

The principal contribution of this study is the detailed physiological characterization of transmyocardial metabolic adaptation during the earliest phases of reperfusion using simultaneous coronary sinus and peripheral blood sampling. Although the cohort size was modest, the prospective acquisition of paired coronary sinus and peripheral samples at multiple predefined reperfusion time points represents a rare and technically demanding dataset in contemporary cardiac surgery research. Direct CS sampling provides a unique window into myocardial-specific metabolic activity that cannot be obtained from systemic biomarkers alone. Accordingly, the principal value of the present study is mechanistic and descriptive, whereas the mortality analyses remain secondary, exploratory, and insufficient to establish the prognostic or clinical utility of RMB. If confirmed in larger and independently validated studies, the RMB framework may provide a practical research tool for integrating routinely available metabolic measurements during early reperfusion. Its reliance on blood gas and lactate analyses could facilitate prospective investigation of reperfusion-related metabolic responses in cardiac surgery.

The present study does not establish clinical thresholds, individual risk estimates, or treatment decisions based on RMB. Its current relevance is therefore primarily mechanistic and hypothesis-generating. Future studies should determine whether RMB provides reproducible information beyond established perioperative variables and whether it can support clinically meaningful risk stratification or targeted management.

Furthermore, integrating multiple metabolic parameters into a single composite index may provide a useful framework for investigating ischemia–reperfusion physiology in other clinical settings, including acute myocardial infarction and cardiac transplantation.

### 5.11. Study Limitations

Several limitations should be acknowledged.

First, this was a single-center exploratory study performed in a relatively small cohort, with only seven in-hospital deaths. Consequently, analyses involving mortality, including ROC-derived AUC estimates, are statistically unstable, likely imprecise, and potentially susceptible to optimism and overfitting. These analyses should therefore be interpreted as descriptive and hypothesis-generating rather than as evidence of established prognostic performance. Validation in substantially larger independent prospective cohorts is required.

Second, the RMB index was derived and evaluated within the same study population. Its equal weighting scheme was selected on physiological and interpretative grounds rather than derived through statistical optimization, and alternative weighting strategies may yield different results. In addition, pH and bicarbonate are physiologically related and may provide partially overlapping information within the composite score. Because RMB also relies on cohort-derived z-score standardization, its values are dependent on the distribution of measurements in the present sample. Evaluation of alternative index-construction methods, and subsequent external validation are therefore required to assess the reproducibility, component redundancy, and generalizability of the framework.

Third, multiple metabolic variables, sampling compartments, time points, and exploratory outcome analyses were examined without a global adjustment for multiplicity. Consequently, the probability of type I error is increased, and some isolated statistically significant findings may represent chance associations. The results should therefore be interpreted according to the consistency, magnitude, and physiological coherence of the observed patterns rather than solely on the basis of nominal *p*-value thresholds. Future confirmatory studies should prespecify a limited number of primary hypotheses and apply an appropriate multiplicity-control strategy.

Fourth, the study population was clinically and procedurally heterogeneous and included several types of valvular interventions as well as a limited number of emergency procedures. Although cardiopulmonary bypass, temperature management, and myocardial protection protocols were standardized, procedure-specific differences in underlying pathology, ventricular remodeling and loading conditions, operative complexity, ischemic duration, and cardiopulmonary bypass duration may have influenced the observed metabolic responses. Formal subgroup analyses were not performed because the procedural categories were small and unbalanced and because only seven deaths occurred. Consequently, the study was insufficiently powered to identify reliable procedure-specific metabolic or outcome associations, and the generalizability of the findings to individual surgical procedures remains uncertain.

Fifth, because of the limited number of outcome events, robust multivariable adjustment was not feasible. Therefore, the incremental prognostic value of RMB beyond established predictors such as EuroSCORE II, cardiopulmonary bypass duration, ischemic time, postoperative troponin release, or low cardiac output syndrome could not be definitively established.

Sixth, although coronary sinus sampling allows direct assessment of myocardial metabolic activity, the strongest prognostic performance was observed for peripheral RMB measured 10 min after reperfusion. This finding suggests that systemic metabolic adaptation may contribute importantly to postoperative outcome and requires further mechanistic investigation.

Finally, the causes of in-hospital mortality were heterogeneous and may not have been exclusively related to myocardial ischemia–reperfusion injury. Therefore, the relationship between RMB and mortality should be interpreted as an association rather than proof of causality.

## 6. Conclusions

This prospective study characterizes the dynamic behavior of lactate, pH, and bicarbonate during myocardial ischemia and early reperfusion using paired coronary sinus and peripheral sampling. The findings provide physiological insight into myocardial and systemic metabolic adaptation following aortic declamping. The RMB framework should be regarded as an exploratory descriptive construct for integrating coordinated metabolic changes rather than as a validated prognostic marker. Its preliminary associations with in-hospital mortality require external validation and confirmation in larger independent prospective cohorts before any clinical or predictive application can be considered.

## Figures and Tables

**Table 6:** Average RMB index.

Mean ± SD	Coronary Sinus	Peripheral
**T1**	−0.01 ± 2.07	−0.007 ± 1.91
**T2**	+0.03 ± 2.27	+0.031 ± 1.94

**Table 7:** Average values of RMB according to the outcome.

Outcome	T1 (CS)	T1 (P)	T2 (CS)	T2 (P)
Alive (n = 94)	0.15 ± 1.98 *	−0.03 ± 1.31	0.16 ± 2.12	−0.10 ± 1.97 *
Deceased (n = 7)	2.54 ± 1.36	−0.10 ± 1.90	−2.33 ± 3.62	2.29 ± 0.87

* *p* < 0.05 compared to alive.

## Data Availability

The original contributions presented in this study are included in the article. Further inquiries can be directed to the corresponding author.
